# Predominance of Biliverdin over Bilirubin in Human Seminal Plasma

**DOI:** 10.3390/biom16040569

**Published:** 2026-04-11

**Authors:** Nina Hojnik, Paola Sist, Sabina Passamonti, Borut Kovačič, Federica Tramer

**Affiliations:** 1 Department of Reproductive Medicine and Gynaecologic Endocrinology, Clinic for Gynecology and Perinatology, University Medical Centre Maribor, 2000 Maribor, Slovenia; borut.kovacic@ukc-mb.si; 2 Faculty of Medicine, University of Maribor, 2000 Maribor, Slovenia; 3 Department of Life Sciences, University of Trieste, 34127 Trieste, Italy; psist@units.it (P.S.); spassamonti@units.it (S.P.); ftramer@units.it (F.T.)

**Keywords:** bilirubin, biliverdin, biosensor, fluorometric analysis, heme metabolism, male infertility, semen, UnaG

## Abstract

Biliverdin (BV) and bilirubin (BR) are established endogenous antioxidants and immune modulators in other organ systems; however, their roles in the male genital tract remain undefined. The aim of this study was to quantify both bile pigments in human seminal plasma using a fluorescent protein biosensor and to examine their associations with basic semen parameters. We analyzed forty-two semen samples from men undergoing infertility evaluation. Biliverdin predominated over bilirubin in 88.1% of samples. Biliverdin concentration ranged from 51.8 to 611.2 nM, whereas bilirubin ranged from 19.7 to 240.7 nM. The mean total amounts per ejaculate were 1054 pmol for biliverdin and 280 pmol for bilirubin. The total amount of bilirubin in the ejaculate was positively correlated with total sperm count (Rs = 0.47; *p* = 0.028), whereas biliverdin showed no significant association (Rs = 0.21; *p* = 0.723). Oligozoospermic samples had significantly lower bilirubin concentrations (*p* < 0.001) and lower total bilirubin amounts (*p* < 0.005). Teratozoospermic samples exhibited significantly higher biliverdin concentrations (*p* < 0.05). This study provides the first simultaneous quantification of biliverdin and unconjugated bilirubin in human seminal plasma and identifies distinct associations with sperm quality. These findings suggest that bile pigments may reflect localized redox-related processes in the male genital tract and may influence male fertility potential.

## 1. Introduction

The prevalence of male infertility is a growing medical problem worldwide [1]. Increasing environmental pressures, such as toxicants or rising temperatures, are associated with declines in human semen quality [2]. Negative environmental factors are often linked to disruption of redox homeostasis [3]. A disturbed balance between oxidants and reductants, referred to as oxidative stress, can be an underlying cause of male infertility [4]. Free radicals formed in the urogenital tract can damage membrane lipids, DNA, and proteins, leading to impaired sperm functionality such as motility and fertilizing ability [5,6]. Of particular concern is that damage to sperm DNA may negatively impact the health of offspring [7,8,9].

Despite this well-established pathophysiological link, routine clinical evaluation of male fertility remains largely limited to conventional semen analysis, which focuses on sperm concentration, motility, and morphology. Beyond spermatozoa, seminal plasma is a biologically active and redox-sensitive fluid that plays an important role in male reproductive physiology. As a composite secretion from the testis, epididymis, and accessory sex glands, seminal plasma reflects the biochemical microenvironments encountered by spermatozoa throughout spermatogenesis, epididymal maturation, and ejaculation [10].

Seminal plasma is not a passive carrier fluid but a dynamic medium enriched with enzymatic and non-enzymatic antioxidants, metal-binding proteins, and redox-active metabolites. These components collectively contribute to redox homeostasis and protection against oxidative injury [11]. Alterations in its biochemical composition have been associated with impaired sperm function and male infertility, even in the absence of overt abnormalities in conventional semen parameters, and have long been recognized as clinically informative [12].

Biochemical profiling of seminal plasma has emerged as a complementary approach to routine semen analysis, particularly in cases where conventional parameters fail to capture underlying metabolic or redox disturbances [13].

This unmet need is especially evident in the context of idiopathic sperm abnormalities. This is a highly prevalent condition characterised by reduced sperm concentration, impaired motility, or abnormal morphology in the absence of identifiable pathological causes [14]. Male infertility is a multifactorial condition influenced by both reversible and irreversible factors. Among these are age, pharmacological treatments, surgical history, exposure to environmental toxins, developmental and genetic background, infections, and systemic diseases [15].

To date, the comprehensive profile of low-molecular-weight redox-active and potentially antioxidant metabolites in the seminal plasma of fertile, subfertile, and infertile men remains incompletely characterised [16]. In this context, targeted biochemical profiling of seminal plasma represents a promising strategy for identifying novel biomarkers and metabolic signatures associated with male reproductive dysfunction.

Among endogenous redox-active molecules, the bile pigments biliverdin and bilirubin have emerged as important modulators of cellular responses related to the regulation of redox balance in various biological systems [17,18,19]. Biliverdin and bilirubin are products of the catabolism of the heme prosthetic group. Although historically regarded as waste products of heme degradation and neurotoxic at extremely high concentrations [20], their intracellular levels are tightly regulated in a tissue-specific manner to maintain redox balance and limit iron-mediated cytotoxicity [21,22].

The role of bile pigments in male reproductive biology remains poorly defined. However, enzymes involved in their metabolism—heme oxygenases (HMOX1 and HMOX2) and biliverdin reductase A (BLVRA)—are highly expressed across multiple compartments of the male reproductive tract (Human Protein Atlas v24.0) [23]. Interest in bile pigments is further supported by evidence linking enzymes responsible for their production to male fertility potential. Heme oxygenase activity has been studied in human seminal plasma and is associated with various forms of male infertility [24,25]. Moreover, HMOX1 functions as a key downstream effector of Nrf2-mediated redox homeostasis, a signalling pathway well established as critical for maintaining oxidative balance in the male reproductive tract [26]. Despite this, the specific functions of biliverdin and bilirubin in reproductive health and disease remain largely unexplored. Characterizing bile pigments in seminal plasma may help to clarify their potential relevance in the redox environment of the male reproductive tract.

A major challenge in studying bile pigments in complex biological fluids such as human seminal plasma has been the lack of sensitive and practical methods for accurately quantifying both biliverdin and bilirubin without matrix interference. Conventional clinical assays, such as the Jendrassik–Grof method, lack sufficient sensitivity to detect low concentrations in complex matrices, while advanced techniques, such as high-performance liquid chromatography, although more accurate, require extraction steps and are impractical for routine use.

In recent years, fluorescent protein-based biosensors have gained an important role in biomedical research. Notably, UnaG, a fluorescent protein derived from the Japanese eel (*Anguilla japonica*), is unique as the only known vertebrate-origin fluorescent protein. UnaG fluoresces upon specific binding to unconjugated bilirubin, offering high sensitivity and specificity [27]. A recombinant fusion protein HUG (HELP + UnaG) combining UnaG with human elastin-like polypeptide was constructed [28]. It enables simultaneous detection of bilirubin and biliverdin through enzymatic conversion by BLVRA and NADPH and has been validated for use in various biological samples [29]. HUG is highly specific and sensitive and can be easily used in complex biological fluids without extraction with organic solvents [30,31]. While not yet commercially available, this emerging technology holds significant promise for future clinical applications.

The aim of the present study was to apply an advanced analytical approach to simultaneously quantify biliverdin and unconjugated bilirubin in human seminal plasma and to explore their associations with basic semen parameters. This work was designed as an exploratory investigation to provide foundational biochemical data and to generate research hypotheses regarding the role of endogenous bile pigments in the redox environment of the male reproductive tract.

## 2. Materials and Methods

### 2.1. Study Population and Ethical Approval

The study included male partners undergoing routine semen analysis for couples’ infertility evaluation at the University Medical Centre Maribor (Maribor, Slovenia). Ethical approval was obtained from the Institutional Review Board (UKC-MB-KME-39/21) and the National Medical Ethics Committee (0120-238/2024-2711-5). All participants provided written informed consent.

Eligible participants were adult men (18–50 years) from couples with a history of infertility, defined as at least 12 months of unsuccessful conception. Samples were collected between June 2022 and June 2023. The analyzed samples included both normozoospermic samples and those with abnormal semen parameters, such as asthenozoospermia (reduced sperm motility), oligozoospermia (low sperm concentration), teratozoospermia (high percentage of sperm with abnormal morphology), or combinations thereof. Semen analysis was performed according to the World Health Organization (WHO) laboratory manual for the examination and processing of human semen (6th ed., 2021) [32]. The results were classified using the 5th percentile reference values for basic semen parameters provided in the manual.

Exclusion criteria comprised obstructive azoospermia, active genitourinary or sexually transmitted infections, and current or recent exposure to hormonal therapy, chemotherapy, or radiotherapy. Additionally, men with extremely low sperm counts (total sperm count < 5 × 10^6^ per ejaculate) were excluded from the analysis. Although information on potential confounders (such as smoking status, alcohol consumption, chronic comorbidities, medication use, and varicocele) was collected, it was not incorporated into the statistical analysis or presented in the manuscript due to the exploratory nature of the study.

### 2.2. Sample Collection, Processing, and Semen Analysis

All samples were provided in the clinic to avoid temperature fluctuations and light exposure and to enable timely analysis. They were obtained by masturbation after 1–10 days of sexual abstinence and allowed to liquefy for 30 min at 37 °C in the dark. An aliquot of native semen was used for standard semen analysis. Basic semen parameters—including volume, pH, sperm concentration, total sperm count, motility, and morphology—were assessed according to WHO guidelines (6th ed., 2021) [32].

The remaining sample was processed within 30–60 min after ejaculation and protected from light throughout, as bile pigments are light sensitive. Spermatozoa and seminal plasma were separated by centrifugation at 800× *g* for 15 min at room temperature. The supernatant (seminal plasma) was aliquoted into sterile tubes and stored at −80 °C for subsequent biochemical analyses.

### 2.3. Reagents

Analytical-grade chemicals were purchased from Merck (Sigma-Aldrich^®^, Merck KGaA, Darmstadt, Germany) and included bilirubin (purity ≥ 99%), biliverdin, human biliverdin reductase A (1500 IU/mL), Dulbecco’s phosphate-buffered saline (PBS), bovine serum albumin (BSA) (purity ≥ 98%), NADPH tetrasodium salt (reduced form), dimethyl sulfoxide (DMSO).

Details regarding HUG (HELP–UnaG) synthesis and purification have been described previously [29].

### 2.4. Measurement of Biliverdin and Bilirubin in Seminal Plasma

The HUG assay has been previously characterized with respect to analytical performance parameters such as linearity, limits of detection and quantification, and potential solvent interference [29]. Accordingly, the objective of the present study was to perform a matrix-specific assessment of assay suitability in human seminal plasma. To this end, validation was limited to fit-for-purpose validation, including spike-and-recovery and storage stability, to evaluate potential matrix effects under the experimental conditions applied.

#### 2.4.1. Spike-and-Recovery Testing

To evaluate the accuracy of bilirubin (BR) and biliverdin (BV) quantification in human seminal plasma using the HUG assay—applied here for the first time—we performed spike-and-recovery experiments. Recovery and matrix effects were evaluated following recommendations for untested sample types [33]. Aliquots of seminal plasma (observed) or PBS with 4 g/L BSA solution, pH 8.5 (expected) (10 µL; *n* = 4) were supplemented with 5 µL of BR or BV standards (10, 7, and 3 µM in PBS with 4 g/L BSA solution, pH 8.5) to achieve low, medium and high increases in endogenous pigment levels. For the analysis within the linearity range of the standard HUG assay calibration (0–50 nM), both seminal plasma and BSA solution were diluted 70-fold with 0.05 g/L HUG. Each aliquot was analyzed in quadruplicate. Data (means ± SD, *n* = 3) were obtained from the net fluorescence of spiked specimens, after subtraction of the basal fluorescence. BR concentrations were determined directly from fluorescence against the standard curve, while BV levels were calculated by subtracting BR from the total BR generated after enzymatic conversion of BV by BLVRA in the presence of NADPH [29].

#### 2.4.2. Stability Testing

In order to evaluate whether it is possible to analyse bile pigments even after their collection and storage at −80 °C, some fresh seminal fluids (*n* = 4) were divided into single-use aliquots immediately after processing. Aliquots were stored at −80 °C and thawed only once at each time point. The bile pigments were measured immediately and after 1 and 6 months to determine their stability over time.

#### 2.4.3. Bile Pigments Quantification

Seminal plasma was thawed and centrifuged at 6000× *g* for 5 min at 4 °C. Bile pigment concentrations were measured using the HUG recombinant fluorescent probe as described previously [29]. Samples were diluted approximately 25-fold, which allowed preservation of analytical sensitivity while maintaining measurements within the calibration range. Samples (80 µL) were diluted to 2 mL of 0.05 g/L HUG solution in PBS pH 8.5 and then divided into 2 aliquots of 0.99 mL each. A volume of 10 µL of PBS was added to the first vial for BR quantification, while 10 µL of BVR mix (final concentrations: 0.05 mM NADPH; 0.187 U/mL BLVRA) was added to the second vial for total conversion of BV into BR, which binds stoichiometrically to HUG. Solutions were dispensed into black 96-well plates (Nunc^®^, Thermo Fisher Scientific, Waltham, MA, USA) and incubated overnight in the dark under ambient conditions at 25 °C, followed by fluorescence measurement (excitation = 485 nm; emission = 535 nm) using a Synergy H1 microplate reader (BioTek, Winooski, VT, USA). Fluorescence signals of bile pigments were corrected for sample autofluorescence by subtracting the fluorescence of the same sample measured in the absence of HUG. Native unconjugated bilirubin concentrations were measured directly. The total BR concentrations were obtained after enzymatic conversion of biliverdin to bilirubin by biliverdin reductase A (BLVRA). Biliverdin concentrations were subsequently calculated as the difference between total and native bilirubin. No negative or near-zero biliverdin values were observed after subtraction. All measurements were performed in duplicate; measurements were considered acceptable when the coefficient of variation (CV) was ≤10%. The emitted fluorescence was quantified by interpolation of a BR standard curve (0–50 nM BR).

### 2.5. Statistical Analysis

Statistical analyses were performed using GraphPad Prism (version 10.5.0; GraphPad Software, San Diego, CA, USA) and Jamovi Desktop (version 2.3; The Jamovi Project, Sydney, Australia). Data distribution was assessed using the Shapiro–Wilk test in combination with visual inspection of Q–Q plots, in accordance with recommended methodological practices.

Descriptive statistics were calculated as mean ± standard deviation (SD) for normally distributed data and as median with interquartile range (IQR) for non-normally distributed data. Observed and expected values in spike-and-recovery and stability experiments were compared using two-way analysis of variance (ANOVA), followed by Šidák’s or Tukey’s post hoc multiple-comparisons tests, as appropriate.

Comparisons between two independent groups were performed using Welch’s *t*-test for normally distributed data with unequal variances or the Mann–Whitney U test for non-normally distributed data. Associations between continuous variables were evaluated using Pearson or Spearman correlation coefficients, depending on data distribution.

*p*-values obtained from pairwise correlation analyses were adjusted for multiple testing using the Benjamini–Hochberg procedure to control the false discovery rate. Statistical significance was defined as a two-tailed *p*-value < 0.05. The selection and application of statistical methods were guided by established principles of biomedical research methodology and data analysis [34].

## 3. Results

### 3.1. Characteristics of the Study Population and Semen Samples

The study included 42 men aged 24–50 years, undergoing evaluation for couple infertility, representing the general clinical population treated at a university-based infertility unit. According to the WHO guidelines (6th ed., 2021) [32], semen samples were classified as normozoospermic or as exhibiting oligozoospermia (≤16 × 10^6^/mL), asthenozoospermia (<30% progressive motility), teratozoospermia (<4% normal forms), or combinations thereof. The exact distribution of samples across semen phenotypes is presented in the supplementary files (Supplementary Table S1). Several potential confounders (such as smoking status, alcohol consumption, medication use, and varicocele) were recorded but were not regarded as exclusion criteria due to the exploratory nature of the study.

The median age of participants was 35.5 years (IQR 32.0–39.8), and the median body mass index (BMI) was 26.9 kg/m^2^ (IQR 24.9–29.1). Semen samples showed considerable inter-individual variability in volume, sperm concentration, total sperm count, motility, morphology, and vitality (Table 1). Abstinence time ranged from 1 to 10 days (median 4 days, IQR 3–5).

### 3.2. Analytical Validation of Bile Pigment Quantification in Seminal Plasma

#### 3.2.1. Spike-and-Recovery Experiments

Spike-and-recovery experiments showed that the HUG assay reliably quantified both bilirubin (BR) and biliverdin (BV) in seminal plasma without detectable matrix interference. As shown in Table 2, no significant differences were found between expected (BSA solution) and measured concentrations at any spike level of BR or BV in seminal plasma (observed). As shown in Table 2, no statistical difference in BR (low: *p* = 0.546; medium: *p* = 0.546; high: *p* = 0.546) or BV (low: *p* = 0.427; medium: *p* = 0.314; high: *p* > 0.999) concentrations were found in seminal plasma (observed) and the BSA solution of all graded additions. These data show no statistically significant differences between expected and measured concentrations under the tested conditions, supporting the preliminary suitability of the assay for use in seminal plasma.

#### 3.2.2. Stability During Storage

**Figure 1:**
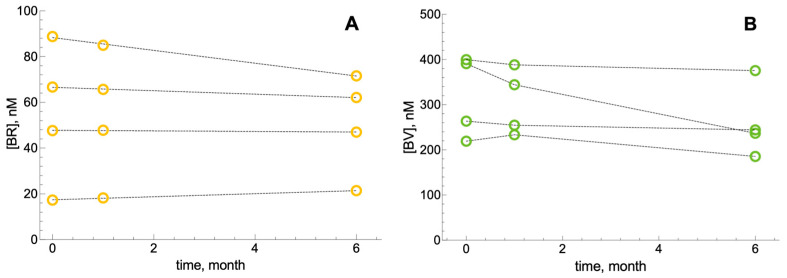
Stability of bilirubin and biliverdin in seminal plasma upon storage. Samples of seminal plasma with variable intrinsic BR and BV concentrations were divided into aliquots and stored at −80 °C. Aliquots were thawed at different times and analyzed for (**A**) BR (*n* = 4) and (**B**) BV (*n* = 4). Each data point represents the mean of three technical replicates. Standard deviation was calculated but is not visible as error bars are smaller than the symbol size.

The stability of BR and BV in seminal plasma stored at −80 °C was assessed over six months. As shown in Figure 1, individual samples (*n* = 4) maintained stable BR concentrations over time (*p* = 0.413). BV concentrations showed greater inter-sample variability but no statistically significant temporal change (*p* = 0.111), indicating adequate stability under the storage conditions used.

### 3.3. Concentration and Distribution of Biliverdin and Bilirubin in Human Seminal Plasma

**Figure 2:**
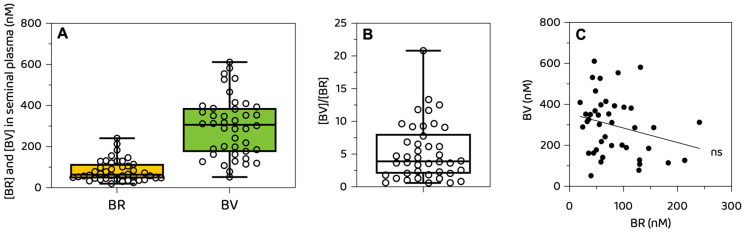
Bilirubin and biliverdin in human seminal plasma. (**A**) BR and BV concentrations in human seminal plasma. (**B**) Ratio between pigments (BV/BR). (**C**) Correlation between BR and BV in seminal plasma (*n* = 42). Note: BV—biliverdin; BR—bilirubin; ns—not significant.

BV and BR were both detectable in all seminal plasma samples, with concentrations spanning a wide range (Figure 2). As shown in Figure 2A, the concentrations of BV (*n* = 42; range—51.8 to 611.2 nM; mean—296.8 nM; median—306.7 nM; SD—144.4; SEM—22.3) and BR (*n* = 42; range—19.7–240.7 nM; mean—82.6 nM; median—63.8 nM; SD—51.3; SEM—7.9) showed substantial inter-individual variability. In the majority (88.1%) of the tested samples, BV exceeded BR (Figure 2B), with a [BV]/[BR] ratio of 5.3 ± 4.4. No statistically significant correlation between BV and BR concentrations was found (Rs = −0.254, *p* = 0.104) (Figure 2C).

### 3.4. Associations Between Bile Pigments and Semen Parameters

The mean total amounts of BV and BR per ejaculate were 1054 pmol and 280 pmol, respectively. The total BR per ejaculate was positively correlated with total sperm count (Rs = 0.47; *p* = 0.028), whereas BV showed no significant correlation (Rs = 0.21; *p* = 0.723). A significant inverse correlation was observed between seminal pH and BV concentration (Rs = −0.47; *p* = 0.028) and total BV amount (Rs = −0.57; *p* = 0.003). Correlations between all measured parameters are presented in Supplementary Table S2, and data distribution is reported in Supplementary Table S3.

**Figure 3:**
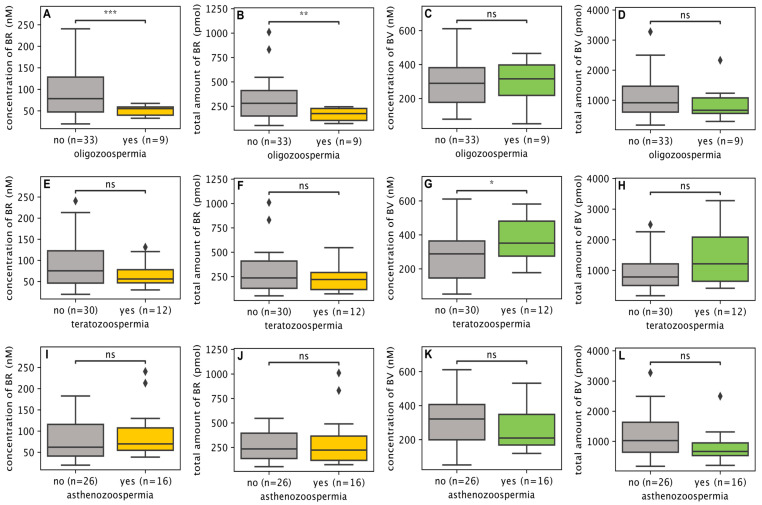
Comparison of bile pigment concentrations (nM) and total amounts per ejaculate (pmol) across semen phenotype groups. The study cohort (Supplementary Table S2) comprised 42 semen samples, including 16 normozoospermic samples, 4 oligozoospermic, 5 teratozoospermic, and 9 asthenozoospermic samples, as well as 7 samples with combined abnormalities. Samples were grouped based on the presence or absence of a given semen abnormality; thus, categories were not mutually exclusive, and samples with combined abnormalities were included in all relevant groups. Accordingly, oligozoospermic samples (*n* = 9; including combined forms) were compared with non-oligozoospermic samples (*n* = 33), teratozoospermic samples (*n* = 12) with non-teratozoospermic samples (*n* = 30), and asthenozoospermic samples (*n* = 16) with non-asthenozoospermic samples (*n* = 26). In oligozoospermic samples, bilirubin (BR) concentration (**A**) and total amount (**B**) were significantly lower compared with non-oligozoospermic samples, whereas no significant differences were observed for biliverdin (BV) (**C**,**D**). In teratozoospermic samples, the BV concentration was significantly higher (**G**), while total amount of BV (**E**) and BR parameters (**F**,**H**) did not differ significantly. No statistically significant differences were identified between asthenozoospermic and non-asthenozoospermic samples for any of the measured parameters (**I**–**L**). Note: BV—biliverdin; BR—bilirubin; *** *p* < 0.001; ** *p* < 0.005; * *p* < 0.05; ns—not significant.

Samples were stratified according to sperm quality categories (oligozoospermia, asthenozoospermia, teratozoospermia, and combinations (Supplementary Table S1). Oligozoospermic samples exhibited significantly lower BR concentrations (*p* < 0.001) (Figure 3A) and total BR amounts in ejaculate (*p* < 0.005) (Figure 3B) compared with non-oligozoospermic samples. No significant differences in BV concentrations or total BV amounts were observed between these groups (Figure 3C,D). In contrast, teratozoospermic samples showed significantly higher BV concentrations (*p* < 0.05) (Figure 3G) compared with non-teratozoospermic samples, while BR concentrations and BR and BV total amounts did not differ significantly (Figure 3E,F,H). BV and BR levels did not differ significantly between asthenozoospermic and non-asthenozoospermic samples (Figure 3I–L). Due to small sample size and overlapping categories, all reported associations are observational and do not imply causality.

## 4. Discussion

This study presents the first quantitative assessment of biliverdin and bilirubin in human seminal plasma. Using the HUG assay, a recombinant UnaG-based biosensor, we simultaneously detected both pigments at nanomolar concentrations without solvent extraction, providing accuracy and reproducibility in a complex matrix. A major advantage of HUG is its high bilirubin affinity, which exceeds that of albumin, allowing the displacement of the pigment from transport proteins and eliminating the need for solvent-based processing [35]. Initial validation of the methodology with spike-and-recovery testing showed no statistically significant differences between expected and measured concentrations under the tested conditions, supporting the suitability of the assay for use in seminal plasma.

The study was designed as an investigation focused on biochemical characterisation rather than functional validation. The simultaneous quantification of biliverdin and unconjugated bilirubin in seminal plasma should therefore be interpreted within a biomarker discovery framework, rather than as evidence of direct antioxidant or immunoregulatory activity. In complex biological systems, descriptive profiling of compartment-specific metabolites is a critical first step towards identifying candidate indicators of local physiological or pathological states. While such approaches do not establish causality, they provide essential groundwork for hypothesis generation and guide the design of subsequent mechanistic and functional studies [36].

A key finding of our study is that biliverdin concentrations exceeded bilirubin concentrations in most of the seminal plasma samples. This pattern differs from that observed in human blood and cerebrospinal fluid by the same analytical method [31], where bilirubin typically predominates, and highlights the tissue-specific nature of bile pigment metabolism. The absence of correlation between biliverdin and bilirubin concentrations further supports the idea that these pigments are not passively derived from plasma filtration but are subject to local regulation within the male genital tract.

The ability to detect associations between bile pigment levels and sperm parameters demonstrates both their biological relevance and the analytical performance of the assay in a complex biological matrix such as seminal plasma. The total amount of bilirubin in the ejaculate was positively correlated with total sperm count, a parameter widely regarded as a robust indicator of male fertility potential reflecting overall spermatogenic output [37]. This association was further supported by subgroup analysis, in which bilirubin levels were significantly reduced in oligozoospermic samples. In contrast, biliverdin showed no significant relationship with sperm concentration or total sperm count.

Biliverdin concentration was elevated in teratozoospermic samples, while bilirubin levels remained unchanged. This dissociation suggests that biliverdin and bilirubin may reflect distinct biological processes. These processes may occur at different stages of spermatogenesis, epididymal maturation, or seminal plasma formation. Comparisons between sperm subgroups should be interpreted with caution due to the small sample sizes and overlap between individual categories. Further studies are needed to define the pathways linking heme degradation to reproductive outcomes in males.

Although pH alone is not regarded as a diagnostic marker of fertility potential and thus not included among the predefined endpoints of this study, additional analysis of additional seminal parameters identified a significant inverse correlation between seminal pH and biliverdin concentration. These findings should be interpreted as hypothesis-generating. Nevertheless, the association may reflect a link between the seminal microenvironment and bile pigment balance. Given that seminal pH is influenced by glandular secretions and metabolic activity, it may indirectly indicate local biochemical conditions relevant to heme metabolism. The potential involvement of enzymatic pathways, including heme oxygenase and biliverdin reductase, remains to be clarified and was not addressed in this study.

By directly quantifying both pigments, the present study provides a new level of detail in assessing heme metabolism in the male reproductive tract. Previous human studies on male infertility have largely focused on heme oxygenase activity in seminal plasma, rather than directly measuring bile pigments [24,25]. Although increased heme oxygenase activity has been reported in infertile conditions, enzyme activity alone does not reflect the downstream balance between biliverdin and bilirubin or their extracellular availability.

The biological interpretation of these findings must, however, remain cautious. Although biliverdin and bilirubin are recognized as endogenous modulators of redox balance and immune signalling in other organ systems, this study does not demonstrate a functional antioxidant or immunoregulatory role for these pigments in semen. No direct markers of oxidative stress, lipid peroxidation, or enzymatic activity were measured, and causal relationships cannot be inferred from the observed associations. The results should therefore be interpreted as indicative of correlations rather than mechanistic effects.

Although data on the relevance of bile pigments in the human male reproductive tract are lacking, animal studies indicate important physiological roles within the epididymal milieu, including microvesicle-mediated transfer of biliverdin reductase A from the epididymal epithelium to the sperm surface [38,39]. Authors have suggested that biliverdin reductase A bound to the sperm surface might protect spermatozoa from oxidative stress, particularly under pro-oxidative conditions in the epididymis.

On the other hand, in teratozoospermia, sperm DNA damage, apoptosis, and oxidative stress are interconnected and represent a common pathogenic mechanism [40]. Stress conditions can trigger upregulation of *HMOX1*, as has been reported in men with varicocele, a condition associated with elevated scrotal temperature and testicular hypoxia [41], which often results in altered sperm morphology. Moreover, experimental studies in animal or cell-line models show that dysregulation of this pathway influences spermatogenesis, steroidogenesis, and germ cell survival under stress conditions [42,43,44]. The role of *HMOX2*, which is highly expressed in late spermatids [23], remains poorly understood. Cell culture data show its involvement in heme homeostasis [45], while rat studies reveal glucocorticoid regulation and tissue-specific transcripts [46]. Chronic restraint stress in rats resulted in overexpression of both *Hmox1* and *Hmox2* in the testis, leading to elevated ROS in semen but not in testicular tissue [47]. Further studies are needed to define the pathways linking heme degradation to the morphological and functional characteristics of spermatozoa and reproductive outcomes in males.

Bile pigments may be candidate extracellular redox-active components within seminal plasma, rather than acting as stoichiometric antioxidants. During the later stages of epididymal transit, spermatozoa lose almost all of their cytoplasm, suggesting that extracellular components, rather than intracellular antioxidants, may be primarily responsible for protection against reactive molecules. A study on goat epididymis found that sperm antioxidant defences decrease during epididymal transit from caput to cauda [48]. Under such conditions, extracellular low-molecular-weight antioxidants present in seminal plasma may contribute to maintaining redox homeostasis. While bilirubin is generally regarded as an antioxidant at the systemic level, its role within the male reproductive tract may differ depending on local conditions [49,50]. When bovine epididymal sperm were challenged with 500 µM H_2_O_2_, both biliverdin and bilirubin counteracted lipid peroxidation, but biliverdin was more effective [39]. The predominance of biliverdin observed in this study may therefore reflect local enzymatic regulation or microenvironmental conditions and it remains unclear whether, or to what extent, biliverdin present in the epididymis is incorporated into or reflected in sperm composition. Progress in this area is further constrained by limited access to human epididymal tissue for research purposes; nevertheless, the central role of the epididymis in sperm maturation and male fertility is well established [51].

Several limitations of this work should be explicitly acknowledged. The cohort size, while appropriate for an exploratory investigation, limits statistical power in subgroup analyses. The abstinence interval varied among participants and may have influenced seminal plasma composition. Abstinence time was reported descriptively and was not included as a covariate in the statistical analysis. Uncontrolled confounders—particularly those related to lifestyle factors—may have influenced the observed findings. As the HUG assay was applied for the first time to human seminal plasma, an initial analytical validation was performed. While the spike-and-recovery approach provides a robust initial assessment of assay performance in biological matrices, further studies across a wider range of sample conditions may help to refine the evaluation of endogenous variability, protein binding dynamics, and matrix complexity. Finally, the cross-sectional design does not allow assessment of temporal dynamics or causal relationships.

Despite these limitations, the study introduces a novel analytical dimension to male reproductive research. The ability to quantify biliverdin and bilirubin simultaneously in seminal plasma provides a new tool for investigating local redox-related processes not captured by conventional semen analysis. While the present findings do not support immediate clinical application, they establish a methodological and conceptual framework for future studies aimed at integrating bile pigment profiling with established oxidative stress markers, functional sperm assays, and longitudinal fertility outcomes.

## 5. Conclusions

This study provides the first simultaneous quantification of biliverdin and bilirubin at nanomolar concentrations in human seminal plasma and examines their associations with sperm parameters. These findings support further investigation of bile pigments as potential indicators of localized redox regulation within the male reproductive tract and highlight the value of biosensor-based approaches for expanding the biochemical characterization of seminal plasma. Despite several limitations, including the relatively small cohort size and sample variability limiting statistical power, particularly in subgroup analyses, this work establishes a foundation for future studies exploring the role of heme catabolism in the regulation of male fertility.

## Figures and Tables

**Table 1:** Basic characteristics of enrolled men and their semen samples (median [IQR]). Each man provided one semen sample.

Characteristics of men involved in the study	Men (*n* = 42)
Age (years)	35.5 (32.0–39.8)
Body mass index (kg/m^2^)	26.9 (24.9–29.1)
**Characteristics of semen samples**	**Semen samples (** ***n* = 42)**
Volume (mL)	3.15 (2.4–4.2)
Abstinence (days)	4 (3–5)
pH	8.2 (7.9–8.4)
Sperm Concentration (×10^6^/mL)	34 (14.4–85.6)
Total sperm count (million)	117.6 (50.4–276.3)
Motility (A + B%)	40.5 (20.3–61.3)
Motility (C%)	4.5 (3.0–9.8)
Motility (D%)	49 (31.3–68.8)
Normal morphology (%)	5 (3–7)
Vitality (%)	81 (74–90)

**Table 2:** Analytical recovery of bilirubin and biliverdin in the human seminal plasma.

	Spike Level	Expected BSA, nM Mean (±SD)	Observed sp, nM Mean (±SD)	Recovery %	*p*-Value
BR	Low	20.4 (±1.7)	18.8(±1.3)	92	0.546
	Medium	47.6 (±4.0)	49.9 (±1.2)	105	0.227
	High	68.5 (±0.5)	71.8 (±1.6)	105	0.221
BV	Low	17.6 (±0.1)	16.9 (±0.3)	96	0.427
	Medium	40.1 (±1.3)	39.3 (±1.5)	98	0.314
	High	53.9 (±0.5)	53.9 (±0.4)	100	>0.999

Note: BR—bilirubin; BV—biliverdin; BSA—bovine serum albumin; sp—seminal plasma; SD—standard deviation.

## Data Availability

The raw data supporting the conclusions of this article will be made available by the authors on request.

## Supplementary Materials

The following supporting information can be downloaded at https://www.mdpi.com/article/10.3390/biom16040569/s1, Table S1. Distribution of samples across subcategories: asthenozoospermia (reduced sperm motility), oligozoospermia (low sperm concentration of sperm in the ejaculate), teratozoospermia (high percentage of sperm with abnormal morphology), or combinations thereof. Table S2. Correlation analysis between basic semen parameters and biliverdin (BV) and bilirubin (BR) in human seminal plasma, expressed as concentrations ([BV], [BR]) and total amounts per ejaculate. Table S3. Data distribution was assessed using the Shapiro–Wilk test and visual inspection of Q–Q plots.

## Abbreviations

The following abbreviations are used in this manuscript:

BV	Biliverdin
BR	Bilirubin
BLVRA	Biliverdin reductase A
HMOX1	Heme oxygenase 1
HMOX2	Heme oxygenase 2
NADH	Nicotinamide adenine dinucleotide
NADPH	Nicotinamide adenine dinucleotide phosphate
UnaG	Naturally occurring protein from the muscle of Japanese eel (*Anguilla japonica*) that emits fluorescence upon binding of bilirubin with high affinity
HELP	Human elastin-like polypeptide
HUG	Acronym of HELP-UnaG
WHO	World Health Organization
BSA	Bovine serum albumin
