# Supplementary material for: Predominance of Biliverdin over Bilirubin in Human Seminal Plasma
Source: Biomolecules. 2026 Apr 11;16(4):569. doi: 10.3390/biom16040569 (PMC13113757; doi:10.3390/biom16040569)
Supplement: Supplementary file 1 [file biomolecules-16-00569-s001.zip › Supplementary data_F_N.pdf]

Table S1. Distribution of samples across subcategories: asthenozoospermia (reduced sperm motility), oligozoospermia (low sperm concentration of sperm in the ejaculate), teratozoospermia (high percentage of sperm with abnormal morphology), or combinations thereof.

**Sample**	**Normo zoospermia**	**Astheno zoospermia**	**Terato zoospermia**	**Oligo zoospermia**
**1**	no	yes	no	no
**2**	no	yes	yes	yes
**3**	yes	no	no	no
**4**	no	no	yes	no
**5**	no	no	no	yes
**6**	no	yes	no	no
**7**	yes	no	no	no
**8**	no	no	yes	yes
**9**	yes	no	no	no
**10**	no	no	yes	no
**11**	no	yes	yes	no
**12**	no	no	yes	no
**13**	yes	no	no	no
**14**	no	yes	no	no
**15**	yes	no	no	no
**16**	yes	no	no	no
**17**	no	no	yes	no
**18**	no	no	yes	no
**19**	no	yes	no	no
**20**	no	yes	yes	no
**21**	yes	no	no	no
**22**	no	yes	yes	yes
**23**	no	no	no	yes
**24**	yes	no	no	no
**25**	yes	no	no	no
**26**	yes	no	no	no
**27**	yes	no	no	no
**28**	yes	no	no	no
**29**	no	yes	yes	no
**30**	no	yes	no	no
**31**	no	yes	no	no
**32**	no	yes	no	no
**33**	yes	no	no	no
**34**	yes	no	no	no
**35**	no	no	no	yes
**36**	no	yes	no	yes
**37**	no	yes	yes	no
**38**	no	yes	no	no
**39**	no	no	no	yes
**40**	yes	no	no	no
**41**	yes	no	no	no
**42**	no	yes	no	no
**sum**	16	16	12	9

Note: Samples can belong to multiple categories simultaneously, so sums exceed total sample size n=42.

Table S2. Correlation analysis between basic semen parameters and biliverdin (BV) and bilirubin (BR) in human seminal plasma, expressed as concentrations ([BV], [BR]) and total amounts per ejaculate. Correlation coefficients (Rs/Rp) were calculated using Spearman or Pearson methods, as appropriate (distribution data is presented in Supplementary Table S3). p-values obtained from pairwise correlation analyses were adjusted for multiple testing using the Benjamini–Hochberg procedure to control the false discovery rate.

Variable 1	Variable 2	n	Correlation (Rs/Rp)	p-value	
**Abstinence (days)**	[BR] (nM)	42	-0.01	0.989	
	[BV] (nM)	42	-0.10	0.884	
	[BR]+[BV] (nM)	42	-0.14	0.850	
	BR total (pmol)	42	0.15	0.850	
	BV total (pmol)	42	0.07	0.884	
	BR+BV total (pmol)	42	0.10	0.884	
**Volume (mL)**	[BR] (nM)	42	-0.06	0.884	
	[BV] (nM)	42	0.14	0.850	
	[BR]+[BV] (nM)	42	0.11	0.884	
**pH**	[BR] (nM)	42	-0.08	0.884	
	[BV] (nM)	42	-0.47	0.028	*
	[BR]+[BV] (nM)	42	-0.47	0.023	*
	BR total (pmol)	42	-0.27	0.472	
	BV total (pmol)	42	-0.57	0.003	**
	BR+BV total (pmol)	42	-0.58	0.003	**
**Morphology (% normal forms)**	[BR] (nM)	42	0.19	0.812	
	[BV] (nM)	42	-0.16 □	0.850	
	[BR]+[BV] (nM)	42	-0.08 □	0.884	
	BR total (pmol)	42	0.17	0.845	
	BV total (pmol)	42	-0.12	0.884	
	BR+BV total (pmol)	42	-0.05	0.884	
**Progressive motility (% A+B)**	[BR] (nM)	42	-0.08	0.884	
	[BV] (nM)	42	0.11	0.884	
	[BR]+[BV] (nM)	42	0.13	0.850	
	BR total (pmol)	42	0.07	0.884	
	BV total (pmol)	42	0.18	0.812	
	BR+BV total (pmol)	42	0.15	0.850	
**Non-progressive motility (% C)**	[BR] (nM)	42	0.08	0.884	
	[BV] (nM)	42	0.04	0.909	
	[BR]+[BV] (nM)	42	0.10	0.884	
	BR total (pmol)	42	0.02	0.966	
	BV total (pmol)	42	0.04	0.909	
	BR+[BV] total (pmol)	42	0.03	0.963	
**Immotile (% D)**	[BR] (nM)	42	0.02	0.966	
	[BV] (nM)	42	-0.23	0.722	
	[BR]+[BV] (nM)	42	-0.27	0.472	
	BR total (pmol)	42	-0.08	0.884	
	BV total (pmol)	42	-0.22	0.722	
	BR+BV total (pmol)	42	-0.19	0.812	
**Sperm concentration (10^6/mL)**	[BR] (nM)	42	0.28	0.472	
	[BV] (nM)	42	-0.18	0.812	
	[BR]+[BV] (nM)	42	-0.07	0.884	
	BR total (pmol)	42	0.32	0.394	
	BV total (pmol)	42	-0.02	0.966	
	BR+BV total (pmol)	42	0.08	0.884	

**Total sperm count (10^6 of sperm)**	[BR] (nM)	42	0.21	0.722	
	[BV] (nM)	42	-0.12	0.884	
	[BR]+[BV] (nM)	42	-0.06	0.884	
	BR total (pmol)	42	0.47	0.028	*
	BV total (pmol)	42	0.21	0.722	
	BR+BV total (pmol)	42	0.32	0.394	
**Progressive motile (A+B) concentration (10^6/mL)**	[BR] (nM)	42	0.19	0.812	
	[BV] (nM)	42	-0.09	0.884	
	[BR]+[BV] (nM)	42	0.00	0.990	
	BR total (pmol)	42	0.29	0.472	
	BV total (pmol)	42	0.07	0.884	
	BR+BV total (pmol)	42	0.14	0.850	
**Immotile (D) concentration (10^6/mL)**	[BR] (nM)	42	0.29	0.472	
	[BV] (nM)	42	-0.17	0.822	
	[BR]+[BV] (nM)	42	-0.06	0.884	
	BR total (pmol)	42	0.25	0.557	
	BV total (pmol)	42	-0.06	0.884	
	BR+BV total (pmol)	42	0.03	0.966	
**Non-progressive motile (C) concentration (10^6/mL)**	[BR] (nM)	42	0.13	0.884	
	[BV] (nM)	42	-0.05	0.909	
	[BR]+[BV] (nM)	42	-0.00	0.990	
	BR total (pmol)	42	0.14	0.850	
	BV total (pmol)	42	0.01	0.986	
	BR+BV total (pmol)	42	0.06	0.884	

Data are presented as correlation coefficients (Rs/Rp). Depending on distribution of data (see Supplementary Data Table S3) Spearman rank correlation was used for all analyses except those marked by ◻ in column Correlation. Statistical significance is indicated as * $p < 0.05$, ** $p < 0.01$, *** $p < 0.001$.

Table S3. Data distribution was assessed using the Shapiro–Wilk test and visual inspection of Q–Q plots. Normally distributed data are presented as mean ± standard deviation (SD), while non-normally distributed data are presented as median and interquartile range (IQR).

Variable	n	Shapiro–Wilk W	p-value	Normal distribution
**Abstinence (days)**	42	0.840	<0.001	No
**Volume (mL)**	42	0.869	<0.001	No
**pH**	42	0.887	<0.001	No
**Morphology (% normal forms)**	42	0.953	0.083	Yes
**Progressive motility (% A+B)**	42	0.931	0.014	No
**Non-progressive motility (% C)**	42	0.876	<0.001	No
**Immotile (% D)**	42	0.940	0.028	No
**Concentration (10^6/mL)**	42	0.891	<0.001	No
**Total sperm count (10^6 of sperm)**	42	0.836	<0.001	No
**Progressive motile (A+B) concentration (10^6/mL)**	42	0.835	<0.001	No
**Immotile (D) concentration (10^6/mL)**	42	0.827	<0.001	No
**Non-progressive motile (C) concentration (10^6/mL)**	42	0.812	<0.001	No
**BR concentration (nM)**	42	0.879	<0.001	No
**BV concentration (nM)**	42	0.964	0.207	Yes
**BR+BV concentration (nM)**	42	0.983	0.792	Yes

**Total amount of BR in ejaculate (pmol)**	42	0.855	<0.001	No
**Total amount of BV in ejaculate (pmol)**	42	0.901	0.002	No
**Total amount of BR+BV in ejaculate (pmol)**	42	0.914	0.004	No

Note: Normality assessed by Shapiro–Wilk test ($p > 0.05$ indicates normal distribution)
